# Is the Surface Anatomy of the Popliteal Crease Related to Lower Extremity Alignment or Knee Osseous Morphology? A Radiographic Study

**DOI:** 10.3390/medicina59101849

**Published:** 2023-10-18

**Authors:** Dong Hwan Lee, Hwa Sung Lee, Bo-Hyoung Kim, Se-Won Lee

**Affiliations:** Department of Orthopedic Surgery, Yeouido St. Mary’s Hospital, College of Medicine, The Catholic University of Korea, 10, 63-Ro, Seoul 07345, Republic of Korea; ldh850606@naver.com (D.H.L.); kuhaha97@gmail.com (B.-H.K.)

**Keywords:** surface anatomy, popliteal crease, knee, limb alignment, osseous anatomy

## Abstract

*Background and objectives:* The popliteal crease varies among individuals, and there has been no prior study on this aspect. We assumed that it may be associated with lower extremity alignment and osseous morphology. To demonstrate this, we conducted a radiographic analysis. *Materials and Methods:* The study was conducted on 121 knees of 63 patients, whose popliteal creases were well distinguished on clinical photographs. PCOA was defined as the angle between the longitudinal axis of the lower leg and the popliteal crease. Through the radiologic examinations performed, the HKA, MPTA, mLDFA, JLCA, MFCA/TEA, and PCA/TEA were measured. Pearson correlation analysis and multiple linear regression analysis were performed on the PCOA and the six radiologic measurements to analyze the relationship. *Results:* Pearson correlation analysis found HKA had the highest coefficient at 0.568. In multiple linear regression, only HKA was associated, excluding all other measurements. *Conclusions:* Popliteal crease obliquity is significantly associated with coronal plane lower extremity alignment and exhibits a stronger correlation than with underlying knee osseous morphology. If future research is conducted based on this, popliteal crease could serve as a valuable clue for predicting lower extremity alignment and the risk of osteoarthritis development.

## 1. Introduction

There are various skin creases in the human body known to develop in the early fetal period [1]. Many studies have been conducted on the palmar crease and wrist crease since they are used as anatomical landmarks in the field of hand surgery [2]. As the most frequently used example, the palmar crease is used for the direct incision or as a surface landmark in trigger finger surgery [3]. Likewise, the wrist crease is used in carpal tunnel release surgery [4]. As such, many studies on their positional relationship with osseous structures as anatomical landmarks have been conducted and utilized [2,5,6]. Itamura et al. conducted a study on the elbow flexion crease and, like the studies on the palmar and wrist creases, they analyzed the positional relationship between the elbow flexion crease and the surrounding osseous structures [7]. There have been only a few studies on the popliteal crease, most of which have been conducted in relation to surgical approaches [8,9,10,11].

We experienced some clinical cases where we observed a change in popliteal crease obliquity in patients with severe arthritis in the medial compartment of the knee after undergoing knee arthroplasty. Therefore, we conducted a study on the biomechanical correlation between popliteal crease obliquity and lower extremity alignment and osseous morphology of the knee joint. As far as we know, there has been no research on whether the popliteal crease reflects osseous anatomy to some extent, aside from studies that have given significance to the popliteal crease as an anatomical landmark for surgery. Studies comparing skin and surface anatomy with osseous morphology are quite rare. There have been studies on the association between earlobe crease shape and cardiovascular events [12], as well as research on the correlation between the length from the elbow to the digit and femur length [13]. However, such studies are scarce and challenging to find in various fields. Furthermore, our study, which directly investigates the relationship with underlying osseous morphology and alignment, is unique. In this regard, we believe that our study holds significant value.

We aimed to demonstrate the association between popliteal crease obliquity and lower extremity alignment and knee osseous morphology. To achieve this, we conducted a radiographic study and formulated two hypotheses for verification. Firstly, we assumed that popliteal crease obliquity is associated with lower extremity alignment and osseous morphology in the coronal plane. To demonstrate this hypothesis, we measured four radiologic parameters in the coronal plane and examined their correlation with popliteal crease obliquity. Secondly, as the popliteal crease is a skin fold that folds during flexion, we hypothesized that popliteal crease obliquity is associated with osseous morphology related to flexion. To demonstrate this hypothesis, we measured two radiologic parameters related to flexion and examined their correlation with popliteal crease obliquity. Based on these investigations, our goal was to identify the primary factors influencing the formation of popliteal crease obliquity for the first time and determine whether popliteal crease obliquity can be used to predict lower extremity alignment and underlying osseous morphology.

## 2. Materials and Methods

### 2.1. Patients

The study was conducted in patients who were admitted to our orthopedic department with knee pain for surgical or conservative treatment between July 2021 and December 2022 and whose popliteal crease was well distinguished on the clinical photograph. Plain radiography was performed, including standing knee extension anteroposterior (AP) view, standing knee 45 flexion posteroanterior (PA) view (Rosenberg view), and standing whole lower limb scanogram, and radiologic measurements were obtained and used for the analysis. Clinical photographs of the popliteal crease were taken for a total of 74 subjects, and 11 of them were excluded from the study. Exclusion criteria: subjects who had already undergone arthroplasty or other knee surgeries and had implants inside, making radiologic measurement difficult, and subjects for whom aforementioned X-rays were taken so inaccurately that radiologic measurement was impossible were excluded from the study. In addition, the subjects whose clinical photographs were not taken accurately enough to clearly identify the popliteal crease were also excluded from the study. This study was approved by the Institutional Review Board of our institution (SC23RISI0004), and all patients provided written informed consent. The analysis of the relationships between popliteal crease obliquity and various radiologic measurements was finally conducted on 121 knees of 63 patients. The patients signed informed consent regarding publishing their data and photographs. The research was performed in accordance with the Declaration of Helsinki.

### 2.2. Popliteal Crease Obliquity Measurement

**Figure 1:**
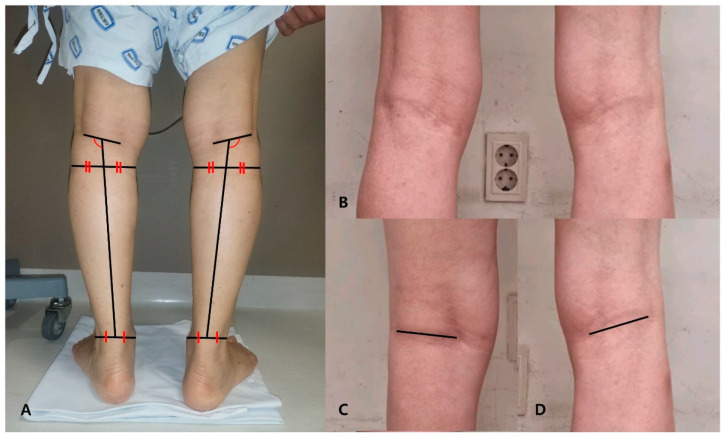
(**A**) Method for measuring the popliteal crease obliquity angle (PCOA). In a clinical photograph of the posterior side of the lower leg, the line connecting the center points of the tibia plateau level and the ankle malleolar level is defined as the lower leg longitudinal axis. The line connecting the two endpoints of the popliteal crease is defined as the reference line. The lateral side angle formed by these two lines is defined as the PCOA. (**B**) A photograph showing the popliteal crease formed in a curvilinear manner. (**C**) Reference line in patients with a straight popliteal crease. (**D**) The reference line is defined as the line connecting the two endpoints in patients with a curvilinear popliteal crease.

With the patient in a standing position, clinical photos of the popliteal crease were taken from the back of the patient. The camera was positioned at knee height, 1 m away from the patient, and the angle of capture was horizontal. The photographs were taken in the hallway of our hospital under indoor fluorescent lighting. The photographs were taken with the legs spread shoulder-width apart, but due to some problems, the widths were not constant, and the popliteal crease obliquity changes greatly due to slight differences in the width. Therefore, the popliteal crease obliquity angle (PCOA) was defined as the angle between the longitudinal axis of the lower leg and the popliteal crease, and the lower leg longitudinal axis was defined as the line connecting the center at the tibia plateau level and the center at the ankle malleolar level (Figure 1A). The shape of the popliteal crease was divided into two main types: a straight line and a curvilinear line (Figure 1B). In the case of a curvilinear popliteal crease, there was a concern that there might be differences in measurement between examiners when measuring the PCOA. To overcome this issue, the line connecting the two end points of the popliteal crease was defined as the standard (Figure 1C,D). Among the angles formed by the lower leg longitudinal axis and the popliteal crease line defined above, the angle of the lateral side obtained and used for the analysis. Two orthopedic surgeons measured the PCOA, and both had sufficient knowledge to measure the angle. For each patient, one of the orthopedic surgeons performed the measurement twice, so a total of three measurements were conducted individually without sharing results. These results were used to check reliability, and the three results were averaged for the final analysis.

### 2.3. Radiologic Measurement

**Figure 2:**
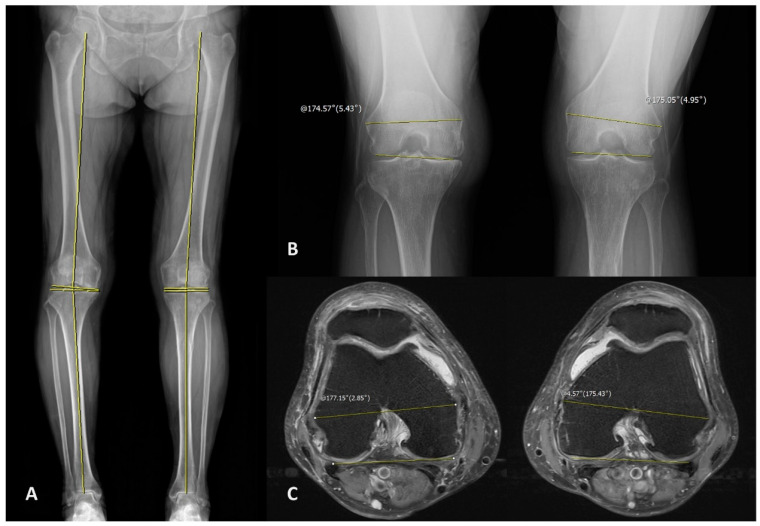
(**A**) The X-ray image with the reference line drawn for measuring the radiologic makers in the standing lower extremity scanogram. (**B**) The X-ray image with the reference line drawn for measuring the mid-flexion condylar axis (MFCA)/transepicondylar axis (TEA) in Rosenberg view. A positive value is assigned when the medial side is open. (**C**) The magnetic resonance imaging (MRI) axial image with the reference line drawn for measuring the posterior condylar axis (PCA)/TEA. A positive value is assigned when the medial side is open.

Several radiologic parameters were measured by standing whole lower limb scanogram, standing knee extension AP, and standing knee 45 flexion PA (Rosenberg view). The scanogram was taken with the patella facing the front in a full weight-bearing state [14]. The Rosenberg view was taken in the PA direction with knee flexion fixed at 45° after weight bearing at 10° feet external rotation, and in the 10° caudal direction from the ground surface [15,16]. For radiologic measurements in X-ray film, the hip-knee-ankle axis (HKA), medial proximal tibial angle (MPTA), mechanical lateral distal femoral angle (mLDFA), and joint-line convergence angle (JLCA) were determined from the standing whole lower limb scanogram (Figure 2A).

The HKA was defined as the angle between the line connecting the femur head center and the tibial spine center and the line connecting the tibial spine center and the center of the talus at the ankle joint [17]. The MPTA was defined as the angle of the medial side between the central axis of the tibia and the proximal tibial joint line, and the mLDFA was defined as the angle of the lateral side between the mechanical axis of the femur and the distal femoral joint line [14,18]. The JLCA was defined as the angle between the proximal tibial joint line and the distal femoral joint line, which is the positive value when the lateral was opened [19]. In addition, the angle between the transepicondylar axis (TEA) which connects both femoral epicondyles and the line connecting the most distal part of both femoral condyles in the Rosenberg view was measured to include radiologic parameters reflecting the mid-flexion state, and this was referred to as the mid-flexion condylar axis (MFCA)/TEA [20,21]. In the MFCA/TEA, the positive value was set in the case where the medial part was opened (Figure 2B).

Many of the patients included in the study underwent magnetic resonance imaging (MRI) of the knee as they were scheduled for arthroplasty, and the MRI images from 69 knees of 49 patients were used for analysis. In patients with MRI, the angle between the TEA and the posterior condylar axis (PCA) in the axial image was determined and named as the PCA/TEA, and the correlation with PCOA was analyzed [22,23]. In the PCA/TEA, the positive value was set in the case where the medial part was opened. The PCA was defined as the line connecting the two points located at the most posterior part of both femoral condyles including the cartilage, to better reflect the state during actual flexion (Figure 2C) [24,25,26]. In this case, it was decided to use the image in which the femoral attachment site of the anterior cruciate ligament starts to be visible for measurement [27]. All radiologic measurements were performed using the PACS software (nU PACS 1.0.0.42.3, TaeYoung Soft Co., Anyang-si, Gyeonggi-do, Korea).

Two fellowship-trained orthopedic surgeons conducted radiologic measurements, and both had sufficient knowledge in measuring angle degrees. Two orthopedic surgeons performed each of the measurements without sharing the results between them, and one of the surgeons obtained two separate measurements, resulting in a total of three measurement results. The results of the measurements were used for the reliability check, and the three results were averaged for the final analysis. The characteristics and measurements of the patient group are shown in Table 1.

### 2.4. Reliability & Statistical Analysis

The intraclass relationship coefficient (ICC) was checked for each parameter in order to check the reproducibility and reliability of the measured popliteal obliquity angles and six radiologic measurements. The ICCs for interobserver variability were 0.989, 0.906, 0.837, 0.956, 0.819, 0.813, and 0.991, respectively, for each of the following parameters: HKA, MPTA, mLDFA, JLCA, MFCA/TEA, PCA/TEA, and the popliteal crease obliquity angle (PCOA). The ICCs for intra-observer variability for the same parameters were 0.983, 0.823, 0.821, 0.976, 0.899, 0.951, and 0.957, respectively (Table 2). SPSS for Windows (SPSS version 26, SPSS Inc., Chicago, IL, USA) was used for the statistical analysis. The Pearson correlation coefficient was calculated to confirm the relationship between the PCOA and 6 radiologic measurements. In addition, we tried to examine the independent variable, that is, the effect of each radiologic parameter on the PCOA, with multiple linear regression analysis. Null hypotheses of no difference were rejected if *p*-values were <0.05.

## 3. Results

In Pearson correlation analysis, the coefficient of correlation of HKA was the highest at 0.568, followed by those of JLCA, mLDFA, MPTA, PCA/TEA, and MFCA/TEA. HKA, JLCA, and mLDFA showed positive correlations with the PCOA, while MPTA, PCA/TEA, and MFCA/TEA showed negative correlations. Of these, the *p*-value of PCA/TEA was 0.058, which was not statistically significant (Table 3). The analysis results of the correlation between radiologic measurements showed that HKA had a significant correlation with all other radiologic measurements. In particular, the highest coefficients of correlation were, in order, JLCA, MPTA, and mLDFA (Table 4). The results of multiple linear regression analysis showed that HKA was the only associated variable, and all other parameters were excluded from the associated variables, indicating no relationship. In the regression analysis, the regression coefficient for HKA was 0.468 (*p*-value = 0.0001), indicating that it showed a significant relationship. The multiple regression equation for PCOA was as follows: PCOA = 104.754 + 0.468 HKA (Table 5). It is considered that JLCA, mLDFA, and MPTA were excluded from the multiple regression analysis as all three measurements were highly correlated with HKA in the Pearson correlation analysis. In addition, MFCA/TEA and PCA/TEA, which were expected to show high degrees of correlation, were shown to have an unexpectedly low correlation in the Pearson correlation analysis and multiple linear regression analysis.

## 4. Discussion

Lower extremity alignment and osseous morphology are very important factors if performing total knee arthroplasty (TKA) or high tibial osteotomy (HTO), both of which are common surgeries for the arthritic knee [28,29,30,31,32]. These are also used as important parameters in the estimation of the prognosis of arthritis [33,34,35]. Therefore, this study was conducted considering that it would be of great significance if popliteal crease obliquity can be used to predict lower extremity alignment and osseous morphology to some extent. This study is more meaningful as no previous study has analyzed the clinical significance of the popliteal skin crease.

The results of Pearson correlation analysis between the popliteal crease obliquity angle and six radiologic measurements showed that HKA, JLCA, mLDFA, and MPTA exhibited high correlations with parameters measured in the coronal plane in order, and exhibited low correlations with MFCA/TEA and PCA/TEA in order. This indicates that alignment or osseous morphology in the coronal plane showed a higher correlation than parameters that reflect flexion. In the multiple linear regression analysis, all other parameters except HKA were excluded, which also indicates that MFCA/TEA and PCA/TEA, the parameters reflecting the flexion state, have little relationship with the PCOA. We assumed that the relationship between flexion-related parameters and the popliteal crease would be strong since the popliteal crease is the line that is formed by folding during flexion, but results differing from the expectation were shown. As most of the patient groups included in this study showed arthritic knees, the range of changes in HKA was relatively large, so there is a possibility that the correlation between the popliteal crease and MFCA/TEA or PCA/TEA was relatively reduced. However, patients with neutral and valgus alignment were also randomly included in this study, and the overall mean HKA was 5.934, suggesting the varus alignment shown in the patients was not so severe. In addition, the total number of knees examined in this study was 121, indicating that the study was conducted with a sufficient number of samples for statistical analysis. That is, the results of the statistical analysis can be considered significant, and it can be concluded that HKA shows the greatest relationship with popliteal crease obliquity, and the effects of the other parameters on the popliteal crease are relatively small.

In the case of mLDFA, MPTA, and JLCA, which were measured on the coronal plane, the value of the other parameter is also determined by HKA if two of the three parameters are determined [32,36]. In other words, HKA and the above three parameters are highly correlated. However, the two parameters mLDFA and MPTA are largely determined at the time of bone formation, so their correlation with each other would be small even considering arthritic changes. Furthermore, no significant correlation between them was shown in the results of the Pearson correlation analysis conducted in this study. The correlation coefficients of PCOA with mLDFA and MTPA were 0.591 and −0.610, respectively. As the difference in the absolute values between them was not large, it could not be considered that one was more correlated than the other. In conclusion, popliteal crease obliquity is considered to be closely related to only HKA, among the six radiologic measurements analyzed in this study.

To summarize, we previously formulated two hypotheses. The first hypothesis was that “popliteal crease obliquity is associated with lower extremity alignment and osseous morphology in the coronal plane”. This was demonstrated since HKA is the most important indicator reflecting coronal plane lower extremity alignment, and its correlation with PCOA was confirmed. However, mLDFA, MPTA, and JLCA did not show significant correlations, indicating that the relationship between coronal plane osseous morphology and popliteal crease obliquity was not established. The second hypothesis was that “popliteal crease, being a skin fold that folds during flexion, is associated with osseous morphology related to flexion”. We examined the correlation of MFCA/TEA and PCA/TEA with PCOA, as indicators reflecting flexion. However, we could not establish this relationship. In summary, this study was unable to establish a clear association between popliteal crease obliquity and underlying osseous morphology. However, it was demonstrated that popliteal crease obliquity is closely associated with whole lower extremity alignment.

**Figure 3:**
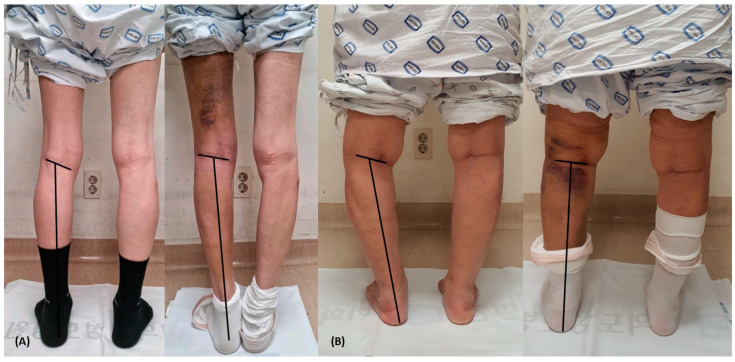
The change in popliteal crease obliquity before and after total knee arthroplasty is shown in the clinical photographs. The black horizontal line is the popliteal crease reference line, and the black vertical line is the lower leg longitudinal axis. (**A**,**B**) are clinical photographs of the same patient, with the left image showing the pre-operative state and the right image showing the post-operative state. Both photographs show that the alignment has been corrected from a varus alignment knee to a neutral alignment knee, and it can be seen that the popliteal crease obliquity has decreased.

Considering that PCOA increased as HKA increased, it is regarded that PCOA increases as arthritic changes in the medial compartment progress in the varus alignment knee. It can be hypothesized that there are three ways in which the popliteal crease can change after it is determined genetically, as a result of arthritic changes and other factors that cause HKA to change. First, a case of moving while forming a circle with a femur mechanical axis from the femoral head center as a radius can be considered. Second, a case of movement that is parallel to the ground surface in the lateral and medial directions according to the changes of varus and valgus can be considered. Lastly, a case of moving while forming a circle with a tibia axis from the talar dome center as a radius can be considered. Based on the results of this study, it can be concluded that the actual movement of the popliteal crease may occur through the first or second manner, as PCOA increases with varus progression and decreases with valgus progression. This is because PCOA is the angle between the lower leg longitudinal axis and the popliteal crease, similar to the angle between the tibia axis and the popliteal crease. It could not be concluded based on the results of this study by which of the two manners the movement of popliteal crease actually occurs. However, the popliteal crease obliquity to the ground surface decreased after TKA in many of the patients included in this study (Figure 3). Therefore, we assume that the acquired change in popliteal crease obliquity is the movement while forming a circle with a femur mechanical axis from the femoral head center as a radius, which is the first manner described above. Further data collection, analysis, and research would be required to conclude this.

The first limitation of this study is that the proportion of patients with varus alignment was high, as most of the patients included in the study were scheduled to undergo arthroplasty due to degenerative arthritis. In this regard, the study was conducted with sufficient numbers of study samples for statistical analysis as mentioned above, so it is considered that there is no problem in checking the clinical significance of popliteal crease obliquity in arthritic patients. The results of this study have limitations affecting their application to the general population, and for this purpose, additional studies to get the data for popliteal crease obliquity and radiologic measurements in the general population would be required. The second limitation of this study is that most of the radiologic measurements analyzed in this study are measurements of parameters that do not have a large scale of the normal range, so the subtle differences that occurred during measurement could affect the results. In order to reduce this deviation, two researchers in this study measured and confirmed the inter-/intra-observer variability using ICC. It is considered that if the study is conducted with a large increase in the total number of patients, the correlation between PCOA and measurements of MPTA or mLDFA, which was not proven in this study, may be verified. Although these limitations exist, this study demonstrated that popliteal crease obliquity in arthritic patients is related to the HKA axis, which has sufficient value as the first clinical study on popliteal skin crease. In addition, if it is possible to estimate lower extremity alignment or predict future changes with the popliteal skin crease based on the evidence of the relationship between PCOA and the HKA axis, it would be possible to use the popliteal skin crease as a diagnostic screening tool for simple self-diagnosis in patients without radiologic examination such as X-ray. This would be a useful tool in modern society where the quality of life is gradually improving and interest in health care is increasing. For this purpose, additional studies on the popliteal skin crease in various races and in the general population would be required.

## 5. Conclusions

We proved that the popliteal crease obliquity angle is closely related to the HKA axis. However, no significant associations were established with mLDFA, MPTA, JLCA, MFCA/TEA, and PCA/TEA. Therefore, it can be concluded that popliteal crease obliquity is significantly associated with coronal plane lower extremity alignment and exhibits a stronger correlation with this than with underlying coronal plane osseous morphology or osseous morphology reflecting flexion. This represents the first study analyzing the shape of the popliteal crease, and it is the first study to analyze which biomechanical factor the popliteal crease is most closely associated with. If future research is conducted based on this, the popliteal crease could serve as a valuable clue for predicting lower extremity alignment and the risk of osteoarthritis development.

## Figures and Tables

**Table 1:** Characteristics and measurements of included patients.

Characteristics	Numbers
Total	Patients: 63/Knee: 121
Gender	Male: 10/Female: 53
Age (mean ± SD)	72.206 ± 9.587 years
Measurements (included numbers)	Mean ± SD (degrees)
HKA (*n* = 121)	5.934 ± 5.853 (positive value when knee is varus alignment)
MPTA (*n* = 121)	85.979 ± 2.493
mLDFA (*n* = 121)	87.784 ± 2.044
JLCA (*n* = 121)	3.748 ± 3.282 (positive value when lateral side is open)
MFCA/TEA (*n* = 121)	5.630 ± 2.096 (positive value when medial side is open)
PCA/TEA (*n* = 69)	5.591 ± 2.220 (positive value when medial side is open)
PCOA (*n* = 121)	107.200 ± 21.254

SD—standard deviation, HKA—hip-knee-ankle axis, MPTA—medial proximal tibial angle, mLDFA—mechanical lateral distal femoral angle, JLCA—joint-line convergence angle, MFCA/TEA—mid-flexion condylar axis/trans-epicondylar axis, PCA/TEA—posterior condylar axis/trans-epicondylar axis, PCOA—popliteal crease obliquity angle.

**Table 2:** Reliability of the evaluated study variables.

Parameter	ICC of Intraobserver (Observer1)	ICC of Interobserver
HKA	0.983 (0.976–0.988)	0.989 (0.977–0.994)
MPTA	0.823 (0.746–0.876)	0.906 (0.865–0.934)
mLDFA	0.821 (0.744–0.875)	0.837 (0.767–0.886)
JLCA	0.976 (0.966–0.983)	0.956 (0.933–0.970)
MFCA/TEA	0.899 (0.855–0.929)	0.819 (0.741–0.874)
PCA/TEA	0.951 (0.921–0.970)	0.813 (0.699–0.884)
PCOA	0.957 (0.906–0.977)	0.991 (0.987–0.994)

Values are expressed as the mean intraclass correlation coefficient (ICC) with 95% CI in parentheses. HKA—hip-knee-ankle axis, MPTA—medial proximal tibial angle, mLDFA—mechanical lateral distal femoral angle, JLCA—joint-line convergence angle, MFCA/TEA—mid-flexion condylar axis/trans-epicondylar axis, PCA/TEA—posterior condylar axis/trans-epicondylar axis, PCOA—popliteal crease obliquity angle.

**Table 3:** Correlations between popliteal crease obliquity angle and radiologic measurements.

Variables	Coefficient of Correlation	*p*-Value
HKA	0.568	0.0001
MPTA	−0.308	0.001
mLDFA	0.369	0.0001
JLCA	0.513	0.0001
MFCA/TEA	−0.226	0.013
PCA/TEA	−0.228	0.058

Pearson’s correlation coefficient. HKA—hip-knee-ankle axis, MPTA—medial proximal tibial angle, mLDFA—mechanical lateral distal femoral angle, JLCA—joint-line convergence angle, MFCA/TEA—mid-flexion condylar axis/trans-epicondylar axis, PCA/TEA—posterior condylar axis/trans-epicondylar axis.

**Table 4:** Correlation between radiologic measurements.

	HKA	MPTA	mLDFA	JLCA	MFCA/TEA	PCA/TEA
HKA		−0.610 (0.0001)	0.591 (0.0001)	0.810 (0.0001)	−0.311 (0.001)	−0.516 (0.0001)
MPTA	−0.610 (0.0001)		−0.171 (0.061)	−0.267 (0.003)	0.046 (0.616)	0.241 (0.046)
mLDFA	0.591 (0.0001)	−0.171 (0.061)		0.377 (0.0001)	−0.145 (0.112)	0.063 (0.605)
JLCA	0.810 (0.0001)	−0.267 (0.003)	0.377 (0.0001)		−0.431 (0.0001)	−0.478 (0.0001)
MFCA/TEA	−0.311 (0.001)	0.046 (0.616)	−0.145 (0.112)	−0.431 (0.0001)		0.646 (0.0001)
PCA/TEA	−0.516 (0.0001)	0.241 (0.046)	0.063 (0.605)	−0.478 (0.0001)	0.646 (0.0001)	

Pearson correlation analysis. Parentheses indicate the level of significance. HKA—hip-knee-ankle axis, MPTA—medial proximal tibial angle, mLDFA—mechanical lateral distal femoral angle, JLCA—joint-line convergence angle, MFCA/TEA—mid-flexion condylar axis/trans-epicondylar axis, PCA/TEA—posterior condylar axis/trans-epicondylar axis.

**Table 5:** Multiple linear regression analysis of the association between dependent variable (popliteal crease obliquity angle) and independent variables (radiologic measurements).

Associated Variable	Regression Coefficient	*p*-Value
HKA	0.468	0.0001
Multiple regression equation	PCOA = 104.754 + 0.468 HKA	
Not associated variables	MPTA, mLDFA, JLCA, MFCA/TEA, PCA/TEA	

HKA—hip-knee-ankle axis, MPTA—medial proximal tibial angle, mLDFA—mechanical lateral distal femoral angle, JLCA—joint-line convergence angle, MFCA/TEA—mid-flexion condylar axis/trans-epicondylar axis, PCA/TEA—posterior condylar axis/trans-epicondylar axis, PCOA—popliteal crease obliquity angle.

## Data Availability

The datasets used in this study are available from corresponding author upon reasonable request.

## Abbreviations

PCOA = popliteal crease obliquity angle, HKA = hip-knee-ankle axis, MPTA = medial proximal tibial angle, mLDFA = mechanical lateral distal femoral angle, JLCA = joint-line convergence angle, MFCA/TEA = mid-flexion condylar axis/trans-epicondylar axis, PCA/TEA = posterior condylar axis/trans-epicondylar axis.
